# Freezing in response to social threat: a replication

**DOI:** 10.1007/s00426-019-01203-4

**Published:** 2019-06-10

**Authors:** Marret K. Noordewier, Daan T. Scheepers, Leon P. Hilbert

**Affiliations:** Faculty of Social and Behavioural Sciences, Social, Economic and Organizational Psychology, Leiden University, PO Box 9555, 2300 RB Leiden, The Netherlands

## Abstract

Freezing is an adaptive defensive response to a stressful event. Recent research suggests that freezing not only occurs in response to physical threats but also in response to social threats (e.g., angry faces; Roelofs et al. in Psychol Sci 21:1575–1581, 2010). Given the practical and theoretical importance of this finding, the current study aimed to replicate and extend it. Following the original study, we measured heart rate while participants viewed emotional faces (angry, happy, neutral). Extending the original study, we included a baseline measure and performed additional, more fine-grained analyses. Our results support the hypothesis that participants show physiological signs of freezing when looking at angry faces. Importantly, we also find this effect when comparing heart rate in the angry block to baseline levels. Interestingly, the heart rate effects are explained by deceleration in the first 30 s of the 1-min angry block, but not in the second 30 s. Like Roelofs et al., we find evidence that the effects are modulated by state anxiety, but our effects are only marginal and we do not replicate the negative correlation between heart rate and state anxiety in the angry block. In general, we thus find evidence for physiological signs of freezing in response to social threat. We discuss implications and venues for future research.

## Introduction

Freezing is a passive, defensive response to a stressful event. It is characterized by reduced body motion, reduced heart rate (bradycardia), and increased muscle tonus (Hagenaars, Oitzl, & Roelofs, 2014a). Freezing is typically associated with fear and is thought to facilitate perceptual and attentional processes aimed at identifying cues for appropriate action (Bradley, Codispoti, Cuthbert, & Lang, 2001; Campbell, Wood, & McBride, 1997; Lang, Davis, & Öhman, 2000). Compared to animal research on freezing (e.g., De Castro Gomes & Landeira-Fernandez, 2008; Kalin & Shelton, 1989; Vianna, Graeff, Brandão, & Landeira-Fernandez, 2001) or human research on other responses to stress such as fight or flight (e.g., Bracha, 2004), human research on freezing is relatively rare.

Recent studies found freezing in response to unpleasant films showing the aftermath of a fatal car accident (Hagenaars, Roelofs, & Stins, 2014b) or pictures of mutilated bodies and corpses (Azevedo et al., 2005; Hagenaars, Stins, & Roelofs, 2012). This latter effect was particularly strong for people who had experienced aversive life events (Hagenaars et al., 2012). Research also showed that experienced fire fighters showed less threat-induced freezing than inexperienced fire fighters, which suggests that people can get used to dealing with threat and probably feel less threatened (Ly, Roijendijk, Hazebroek, Tonnaer, & Hagenaars, 2017). At the same time, reduced freezing has also been found in people with PTSD and borderline personality disorder (Adenauer, Catani, Keil, Aichinger, Neuner, 2010; Fragkaki, Roelofs, Stins, Jongedijk, & Hagenaars, 2017; Stoffels, Nijs, Spinhoven, Mesbah, & Hagenaars, 2017), which is likely explained by increased hyperarousal and vigilance that tends to attenuate freezing (see also Ly et al., 2017).

Apart from arising in response to physical stressors, freezing can also occur in response to social threats. Specifically, Roelofs, Hagenaars, and Stins (2010) showed freezing in response to angry faces, suggesting that freezing is a generic threat response (see also Hagenaars et al., 2014a, b). This is important because freezing in response to social situations has several practical implications ranging from the treatment of psychological trauma (Kozlowska, Walker, McLean, & Carrive, 2015) to improving the quality of social interactions (Mendes, Blascovich, Hunter, Lickel, & Jost, 2007). Given the important theoretical and practical consequences of freezing in response to social stress, together with the fact that Roelofs et al. (2010) is the only study showing freezing in response to social threat, the current study aimed to replicate Roelofs et al. (2010).

In the study by Roelofs and colleagues (2010), participants were presented with emotional (angry, happy, neutral) faces while participants stood on a stabilometric force platform that registered body sway (see also Ly, Huys, Stins, Roelofs, & Cools, 2014). While watching the faces, heart rate was measured. Participants viewed pictures of emotional faces in 1-min blocks that showed angry, happy, or neutral expressions. The angry faces were an operationalization of social threat as they communicate dominance and tend to induce fear (Öhman & Mineka, 2001). In line with predictions, participants showed less body sway when looking at the angry faces as compared to the neutral and happy faces. This effect was most pronounced for people high in state anxiety, as measured with the State-Trait Anxiety Inventory (STAI; Spielberger, Gorsuch, Lushene, Vagg, & Jacobs, 1983). Similarly, there was a marginal effect of emotional face on heart rate, which became significant when state anxiety was added as a covariate to the analyses. Subsequent analyses showed that heart rate during the angry block was lower than during the happy block. Moreover, state anxiety correlated negatively with heart rate in the angry block, but not in the happy block.

We consider it to be important to replicate this study, to establish the robustness and validity of the effect. Roelofs et al. (2010) conducted a single study and particularly the heart rate findings are important to replicate given that the effect of emotional face only became statistically significant when using the STAI as a covariate, which was not the case for the body sway findings (see also Simmons, Nelson, & Simonsohn, 2011). Moreover, the analyses of heart rate focused on the angry and happy blocks alone and excluded the neutral block. Establishing the robustness of the heart rate effect is especially important as changes in body sway alone, without heart rate reduction, can also be explained by attention processes, rather than by freezing (Hagenaars et al., 2014a, b, see also Bradley, 2009; Perakakis et al., 2012).

Our study was—as much as possible—a direct replication of the Roelofs et al. (2010) study, with the following four modifications: First, we focused on heart rate and did not include a measure of body sway. Second, unlike the original study, we also included males in our sample. Third, participants were videotaped with a webcam during the study (see “Method” for more details). Finally, we included a baseline measure of heart rate, which allowed us to compare the effects of looking at emotional faces to a neutral state, in addition to comparing the different emotion blocks to each other.

This latter addition is important as research suggests that neutral facial expressions tend to be perceived as slightly negative rather than as completely neutral (e.g., Arce, Simmons, Stein, Winkielman, Hitchcock, & Paulus, 2009; see also Somerville, Kim, Johnstone, Alexander, & Whalen, 2004). When the neutral block is not perceived to be neutral but instead as slightly negative, this could lower the chances of finding differences in heart rate between angry and neutral faces, which could explain why Roelofs et al. (2010) did not find a significant difference in heart rate between the angry and the neutral block.

Apart from these changes to the procedure, we also extended the statistical analyses, compared to those reported by Roelofs et al. (2010). Following Roelofs et al. (2010), we first tested whether looking at angry faces results in a lower heart rate than looking at neutral or happy faces, and whether this is particularly the case for people who report high state anxiety. Second, we also compared heart rate during the blocks to a baseline measure of heart rate, as well as to heart rate just before the block when participants looked at a fixation cross (see “Method” for details). We included the second comparison to rule out any order effects (e.g., a lower heart rate due to relaxation at the end vs. beginning of the study). Finally, we also examined heart rate within four 15-s epochs within each block. We reasoned that when people look at angry faces, they might engage in emotion regulation to reduce the threat (Hartley & Phelps, 2010), which would result in stronger effects in the first epochs as compared to the later epochs.

We report all manipulations, all measures, and all data exclusions.

## Method

### Participants and design

A total of 60 Dutch students were recruited to participate in exchange for course credit or a monetary reward. The data of six participants were excluded from analyses: Two participants aborted the experiment because they did not feel well; for two participants, the heart rate signal was too distorted to analyze; the data of one participant were not recorded due to a technical error; finally the data of one participant were excluded because she indicated that she anticipated the possible appearance of sharks in the aquatic movie we played during the baseline (see below), which could have potentially increased her heart rate. This resulted in a final sample of 54 participants (44 females, 10 males; *M*_age_ = 22.56, *SD*_age_ = 2.93). All participants had normal or corrected-to-normal vision.

The study had a within-subjects design, where after a baseline measure, emotional faces (angry, happy, neutral) were delivered to the participants in a random order. Heart rate was measured as dependent variable, and state anxiety (STAI; Spielberger et al., 1983) was measured as a covariate.

### Procedure and materials

Upon arriving in the laboratory and after signing the informed consent, the experimenter attached the electrodes to the participants’ wrists and ankles following a Lead 1 configuration. Participants were then led to an individual cubical where the electrodes were attached to the ECG100c module of a Biopac MP150 system. Heart rate was recorded using AcqKnowledge software and analyzed in Matlab using the “Physiodata toolbox” (see https://physiodatatoolbox.leidenuniv.nl).

Participants were instructed to stand at marks on the floor, which was approximately at a 1-m distance from the computer screen that was adjusted in height to fit the participants’ eye-level. We asked participants to stand during the experiment, to mimic Roelofs et al.’s (2010) procedure, where participants stood on a balance board. During the study, participants were videotaped using a webcam that was placed right under the computer screen. The video recordings were made to assess the reliability of a new remote photo-plethysmographic measure (i.e., a heart rate measure based on skin color variation, see e.g., Allen, 2007). This was a beta-version of a new software tool, which in its current form and setting appeared to be unreliable and the data will not be discussed further.

In the first part of the experiment, participants watched a 5-min aquatic video showing a coral reef. This was intended to make participants feel at ease and to create a baseline recording of the participants’ heart rate. As a baseline, we used the heart rate during the last minute of the movie. After the baseline, the experimental phase started. We used the same 20 pictures as Roelofs et al. (2010) depicting 20 models (ten females, ten males) of the Karolinska Directed Emotional Faces Database (Lundquist, Flykt, & Öhman, 1998; see Roelofs et al., 2010, for picture codes). The pictures were frontal portraits of the models showing angry, happy, and neutral  expressions, leading to a total of 60 stimuli. Replicating the procedure of the original study, and to reduce distraction from the facial expression, we cropped the faces and displayed them against a black background. The pictures were presented block-wise in a random order (angry, happy, neutral), with a pause of 7 s between blocks and 2 additional block-to-block transition seconds, where a fixation cross appeared in the middle of the screen. Each picture was presented for 3 s, leading to a total block length of 60 s. Given the very strong effect of the stimuli on pleasantness ratings in the manipulation check of the original study (*F*[2,45] = 983.6, *p* < .01, $\eta_{{\text{p}}}^{2}$ = .98, Roelofs et al., 2010), we omitted this manipulation check as we were confident that also in the current study the stimuli would differ in perceived pleasantness in the intended direction. Note that because the pleasantness ratings were at the end of the original study, the decision to omit this part did not change the setup of the main part of the current study.

Following the block-wise stimulus presentation, and similar to Roelofs et al. (2010), participants completed the Dutch version of the state-subscale of the Spielberger State-Trait Anxiety Inventory (STAI, Spielberger et al., 1983). The state-subscale of the STAI consists of 20 self-report items, measuring how anxious participants feel at the moment (e.g., “I feel nervous”), on a 4-point scale, ranging from 1 = not at all to 4 = very much so (*α* = .94). Then, participants also reported their age and gender.

At the end of the study, participants were fully debriefed and the experimenter entered the cubicle to detach the electrodes. Finally, participants were compensated and thanked for their participation.

## Results

First, we replicated the analyses reported by Roelofs et al. (2010), i.e., we compared heart rate in beats-per-minute between blocks, without taking the baseline into consideration (step 1). Second, we compared heart rate during the blocks to baseline levels of heart rate, as well as to the moment just preceding the block (step 2). Finally, we tested how heart rate develops over time within each block (i.e., whether heart rate is particularly low in the beginning of the angry block or whether lowered heart rate lasts the entire block; step 3).

Before running the analyses, we checked for outliers (defined as higher than three standard deviations above or below the mean). We first report all analyses with the entire sample, i.e., including (potential) outliers. Then, when outliers are present, we subsequently report the respective analysis without the outlier(s).

### Step 1: Heart rate comparisons between blocks

In the first step, we performed the same analyses as in Roelofs et al. (2010) and compared the blocks to each other using a repeated-measures ANOVA with block (angry, happy, neutral) as within-subjects factor. After that, we included standardized state anxiety as a covariate to the model.

Analyses on the entire sample showed no effect of block, Wilks’ lambda = .93, *F*(2,52) = 2.10, *p* = .132, $\eta_{{\text{p}}}^{2}$ = .08. Adding standardized state anxiety (STAI) as a covariate showed a marginal effect of block, Wilks’ lambda = .90, *F*(2,51) = 2.80, *p* = .070, $\eta_{{\text{p}}}^{2}$ = .10, but no block × STAI interaction, Wilks’ lambda = .94, *F*(2,51) = 1.79, *p* = .178, $\eta_{{\text{p}}}^{2}$ = .07. Contrast analyses showed that heart rate in the angry block (*M* = 92.19, *SD* = 16.10) was lower than heart rate in both the neutral (*M* = 93.53, *SD* = 16.50) and the happy blocks (*M* = 93.27, *SD* = 15.89), *F*(1,52) = 4.47, *p* = .039, $\eta_{{\text{p}}}^{2}$ = .08, and *F*(1,52) = 4.25, *p* = .044, $\eta_{{\text{p}}}^{2}$ = .08, respectively.

Results were highly similar when we excluded one participant who had an extreme high STAI score (*Z* = 3.43). Again, the ANOVA did show no overall effect of block, Wilks’ lambda = .93, *F*(2,51) = 1.87, *p* = .164, $\eta_{{\text{p}}}^{2}$ = .07, but a significant effect of block when the STAI was added as a covariate, Wilks’ lambda = .86, *F*(2,50) = 4.23, *p* = .020, $\eta_{{\text{p}}}^{2}$ = .15. In addition, there was a marginal block × STAI interaction, Wilks’ lambda = .89, *F*(2,50) = 3.11, *p* = .053, $\eta_{{\text{p}}}^{2}$ = .11. Contrast analyses showed that the heart rate in the angry block (*M* = 92.33, *SD* = 16.22) was lower than heart rate in the neutral (*M* = 93.61, *SD* = 16.65) and happy blocks (*M* = 93.38, *SD* = 16.03), *F*(1,51) = 7.13, *p* = .010, $\eta_{{\text{p}}}^{2}$ = .12, and *F*(1,51) = 5.84 *p* = .019, $\eta_{{\text{p}}}^{2}$ = .10, respectively. Subsequent correlation analyses showed no significant correlations between heart rate and STAI in any of the blocks (*r*_angry_ = .16, *p* = .253; *r*_neutral_ = .07, *p* = .638; *r*_happy_ = .07, *p* = .621).

Together, these results show that heart rate in the angry block is lower than the heart rate in the neutral and happy blocks. Like Roelofs et al. (2010), we find a modulation by state anxiety, but in the current study this is only marginal and we do not replicate the negative correlation between heart rate and STAI. Moreover, this modulation only occurs when we exclude a STAI outlier.

### Step 2: Comparing blocks with baseline and pre-block heart rate

In the second step, we included baseline heart rate as a comparison in the analyses and conducted a repeated-measures ANOVA on heart rate with block (baseline, angry, happy, neutral) as a within-subjects factor. After this, we repeated the analyses but using the 2-s pre-block heart rate as a comparison.

*Baseline comparison*. Analyses on the entire sample showed a marginal effect of block, Wilks’ lambda = .86, *F*(3,51) = 2.71, *p* = .055, $\eta_{{\text{p}}}^{2}$ = .14. Contrast analyses showed that heart rate was lower during the angry block (*M* = 92.19, *SD* = 16.10), than during baseline (*M* = 94.17, *SD* = 16.38), *F*(1,53) = 7.67, *p* = .008, $\eta_{{\text{p}}}^{2}$ = .13. Additionally, the heart rate during the happy block (*M* = 93.27, *SD* = 15.89) was marginally lower than baseline level, *F*(1,53) = 3.07, *p* = .086, $\eta_{{\text{p}}}^{2}$ = .06. Heart rate during the neutral block (*M* = 93.53, *SD* = 16.50) did not differ from baseline, *F*(1,53) = 1.15, *p* = .288, $\eta_{{\text{p}}}^{2}$ = .02.

Results were again highly similar when we excluded one participant who had an extremely high baseline heart rate (*Z* = 3.00). When excluding this participant from the analysis, there was a marginal effect of block, Wilks’ lambda = .88, *F*(3,50) = 2.32, *p* = .086, $\eta_{{\text{p}}}^{2}$ = .12. Heart rate was lower during the angry block (*M* = 91.53, *SD* = 15.49), than during baseline (*M* = 93.25, *SD* = 15.04), *F*(1,52) = 6.43, *p* = .014, $\eta_{{\text{p}}}^{2}$ = .11. Heart rate during the neutral block (*M* = 92.70, *SD* = 15.48) nor during the happy block (*M* = 92.54, *SD* = 15.13) differed significantly from baseline levels, *F*(1,52) = .82, *p* = .370, $\eta_{{\text{p}}}^{2}$ = .02, and *F*(1,52) = 2.10, *p* = .154, $\eta_{{\text{p}}}^{2}$ = .04, respectively. Thus, these additional analyses, where we compared heart rate in response to the stimuli to a state of rest, revealed some evidence for a physiological correlate of freezing in responses to socially threatening stimuli.

*Pre-block comparison*. To rule out any order effects (e.g., effects could be partially explained by a lower heart at the end vs. beginning of the study), we used heart rate when participants watched the 2-s fixation cross just before a block as a comparison for heart rate during this respective block. Results showed that during the angry block, heart rate was marginally lower than during the 2 s just preceding the block (*M*_pre-angry_ = 94.07, *SD* = 16.48), Wilks’ lambda = .95, *F*(1,53) = 3.01, *p* = .089, $\eta_{{\text{p}}}^{2}$ = .05. There were no differences for the neutral block (*M*_pre-neutral_ = 94.86, *SD* = 17.45) and the happy block (*M*_pre-happy_ = 94.61, *SD* = 16.71), Wilks’ lambda = .97, *F*(1,53) = 1.73, *p* = .194, $\eta_{{\text{p}}}^{2}$ = .03, and Wilks’ lambda = .96, *F*(1,53) = 2.15, *p* = .149, $\eta_{{\text{p}}}^{2}$ = .04, respectively. Thus, with this more direct comparison, the effects generally replicate although they tend to become somewhat weaker.

### Step 3: Heart rate comparisons within blocks

Finally, we analyzed the temporal unfolding of heart rate within the angry block, to better understand whether heart rate deceleration occurs during the entire block or whether it is particularly pronounced at the beginning of the block. We reasoned that it is possible that people habituate to looking at angry faces and that therefore the effects are particularly strong at the beginning of the angry block. To test this, we computed the beats per minute for the angry block in 15-s epochs (i.e., Epoch 1: seconds 0–15; Epoch 2: seconds 15–30; Epoch 3: seconds 30–45; Epoch 4: seconds 45–60). Even though we did not expect to find any effects for the happy and the neutral block, we conducted the same analyses for these blocks as well. We conducted repeated-measures ANOVAs with baseline and the four 15-s epochs within each block separately, and when there was an effect of epoch, we compared the epoch means to the baseline with within-subjects contrasts (see Table 1 for means and SDs). After that, we repeated these analyses with the 2-s pre-block heart rate as a comparison.

**Table 1:** Mean heart rate (*SD*) as a function of block, separated for each 15-s epoch

Block	Seconds 0–15	Seconds 15–30	Seconds 30–45	Seconds 45–60
Angry	90.97^b^ (16.51)	91.51^b^ (16.86)	92.66^a^ (15.65)	93.59^a^ (16.26)
Happy	92.63^a^ (17.36)	93.05^a^ (16.07)	93.35^a^ (16.15)	93.80^a^ (15.99)
Neutral	92.87^a^ (17.22)	93.00^a^ (16.32)	94.01^a^ (16.69)	94.25^a^ (16.61)

Means with different subscripts in rows differ in within-subjects contrasts from baseline (i.e., *M* = 94.17^a^, *SD* = 16.38) at *p* < .05 in within-subjects contrasts. Analyses reported include the baseline outlier; results excluding this participant are the same

*Baseline comparison*. In the angry block, there was an effect of epoch, Wilks’ lambda = .76, *F*(4,50) = 4.06, *p* = .006, $\eta_{{\text{p}}}^{2}$ = .25. Within-subjects contrasts comparing baseline to each epoch showed that heart rate in the first two epochs of the angry block was significantly lower than heart rate during the baseline: seconds 0–15, *F*(1,53) = 12.99, *p* = .001, $\eta_{{\text{p}}}^{2}$ = .20, seconds 15–30, *F*(1,53) = 1.82, *p* = .002, $\eta_{{\text{p}}}^{2}$ = .17. Moreover, the heart rate in the third epoch was marginally lower than baseline: seconds 30–45, *F*(1,53) = 3.88, *p* = .054, $\eta_{{\text{p}}}^{2}$ = .07. Finally, the heart rate in the last epoch did not differ from baseline: seconds 45–60, *F*(1,53) = .60, *p* = .443, $\eta_{{\text{p}}}^{2}$ = .01. These results show that heart rate deceleration in the angry block is particularly strong and reliable in the first half of the block and levels off during the second half of the block. As anticipated, in the neutral and the happy blocks, no effects of epoch were found, Wilks’ lambda = .89, *F*(4,50) = 1.59, *p* = .192, $\eta_{{\text{p}}}^{2}$ = .11, and Wilks’ lambda = .93, *F*(4,50) = .92, *p* = .460, $\eta_{{\text{p}}}^{2}$ = .07, respectively.

Results were similar when excluding the participant with the extremely high baseline heart rate (see above). In the angry block, there was an effect of epoch, Wilks’ lambda = .77, *F*(4,49) = 3.71, *p* = .010, $\eta_{{\text{p}}}^{2}$ = .23. Heart rate in the first two epochs was significantly lower than heart rate during the baseline: seconds 0–15, *F*(1,52) = 11.62, *p* = .001, $\eta_{{\text{p}}}^{2}$ = .18, seconds 15–30, *F*(1,52) = 9.49, *p* = .003, $\eta_{{\text{p}}}^{2}$ = .15. The third epoch was marginally lower than baseline: seconds 30–45, *F*(1,52) = 2.88, *p* = .096, $\eta_{{\text{p}}}^{2}$ = .05. The last epoch did not differ from baseline: seconds 45–60, *F*(1,52) = .18, *p* = .673, $\eta_{{\text{p}}}^{2}$ = .003. In the neutral and the happy blocks, no effects of epoch were found, Wilks’ lambda = .90, *F*(4,49) = 1.39, *p* = .251, $\eta_{{\text{p}}}^{2}$ = .10, and Wilks’ lambda = .95, *F*(4,49) = .68, *p* = .611, $\eta_{{\text{p}}}^{2}$ = .05, respectively.

*Pre-block comparison*. Next, we repeated these epoch analyses using the 2-s pre-block heart rate as a comparison. Similar to the analyses involving the baseline measurement, results showed an effect of epoch for the angry block, Wilks’ lambda = .77, *F*(4,50) = 3.66, *p* = .011, $\eta_{{\text{p}}}^{2}$ = .23. Heart rate was significantly lower than the pre-block heart rate in the first two epochs: seconds 0–15, *F*(1,53) = 7.07, *p* = .010, $\eta_{{\text{p}}}^{2}$ = .12, seconds 15–30, *F*(1,53) = 4.40, *p* = .041, $\eta_{{\text{p}}}^{2}$ = .08. However, the heart rate in the third and fourth epochs was not lower than pre-block heart rate: seconds 30–45, *F*(1,53) = 2.01, *p* = .162, $\eta_{{\text{p}}}^{2}$ = .04, and seconds 45–60, *F*(1,53) = .17, *p* = .686, $\eta_{{\text{p}}}^{2}$ = .003, respectively. Thus, also when comparing the angry block to the 2-s pre-block heart rate, heart rate deceleration is particularly strong and reliable in the first half of the block.

Next, we explored the effects of epoch for the neutral and the happy block. For the happy block, there was again no effect of epoch, Wilks’ lambda = .87, *F*(4,50) = 1.85, *p* = .134, $\eta_{{\text{p}}}^{2}$ = .13. For the neutral block, a marginal effect of epoch appeared, Wilks’ lambda = .84, *F*(4,50) = 2.34, *p* = .068, $\eta_{{\text{p}}}^{2}$ = .16. The heart rate in the first two epochs of the neutral block were (marginally) lower than the pre-block heart rate: seconds 0–15, *F*(1,53) = 4.24 *p* = .044, $\eta_{{\text{p}}}^{2}$ = .07; seconds 15–30, *F*(1,53) = 3.35, *p* = .073, $\eta_{{\text{p}}}^{2}$ = .06, respectively. The heart rate during seconds 30–45 and seconds 45–60 did not differ from the pre-block heart rate, *F*(1,53) = .55, *p* = .463, $\eta_{{\text{p}}}^{2}$ = .01, and *F*(1,53) = .28, *p* = .598, $\eta_{{\text{p}}}^{2}$ = .005, respectively. These results thus show a trend in heart rate deceleration for the beginning of the neutral block. One explanation for this finding could be that neutral facial expressions are perceived as slightly negative, as also alluded to in the Introduction (e.g., Arce et al., 2009; Somerville et al., 2004).

## Discussion

The aim of this study was to replicate and extend the findings of Roelofs et al. (2010), who were the first to show a human freezing response to social threat. That is, Roelofs et al. showed less body movement and lower heart rate in response to angry faces, which was particularly the case for participants who scored high on state anxiety. The original study was highly important, because it showed that freezing is not restricted to physical threats alone (Hagenaars et al., 2012, 2014a, b), but represents a more generic stress response to a variety of threatening stimuli.

We deemed it important to replicate the heart rate findings as the original results were marginal and only emerged after adding state anxiety as covariate. Given that heart rate is a key component of the freezing response (Hagenaars et al., 2014a, b), it is important to establish the robustness of this effect. The setup of our study was similar to the original study with two important additions: We added a baseline measure to be able to compare the heart rate effects to a neutral state and to rule out potential threat effects in the neutral block (Arce et al., 2009; Somerville et al., 2004). Moreover, we also tested whether freezing would be particularly strong at the beginning of the block, by analyzing heart rate in 15-s epochs in addition to the full 1-min blocks.

Our results support the hypothesis that participants show a decrease in heart rate when looking at angry faces, which is in line with a freezing response. Importantly, we also find this when comparing the heart rate in the angry block to a baseline measure. Interestingly, our 15-s epoch analyses showed that the heart rate effects are explained by an effect in the first half of the block. Like Roelofs et al. we find that the block effects are modulated by state anxiety, although only when excluding a STAI outlier. The fact that we only find this modulation when excluding an STAI outlier might suggest that the effects are not extremely reliable, as individual data-points can then have a substantial influence on effects. Indeed, in our study, the effect is marginal and we do not replicate the negative correlation between heart rate and STAI in the angry block.

While our findings are not completely in line with Roelofs et al. (2010), they support the notion that people show physiological signs of freezing in response to social threat. Moreover, the finding that the effects are most pronounced in the beginning of the block may suggest that after about half a minute people got used to looking at angry faces, resulting in habituation in their physical responses (Foa & Kozak, 1986). An alternative explanation would be that participants engage in emotion regulation processes that helped them lower the threat of angry faces (Hartley & Phelps, 2010). Future research could further explore the possibility of regulation and/or habituation in the context of freezing in response to social threat.

There are other venues for future research—both fundamental and applied—on freezing responses to social threats. A basic question concerns how the freezing response relates to other, more active, responses to social stressors that are characterized by an increase in heart rate (see also the defense cascade model in e.g., Lang, Bradley, & Cuthbert, 1997; and the connection between freezing and action preparation in Gladwin, Hashemi, van Ast, & Roelofs, 2016; Roelofs, 2017). Particularly interesting in this context are the cardiovascular response patterns indicative of “challenge” and “threat” motivational states (Blascovich & Mendes, 2010). On the one hand, it seems likely that in many stressful situations, a freezing response precedes a more active threat response later on. On the other hand, since freezing helps to identify appropriate actions to deal with the stressor, it may lead either to stronger threat or stronger challenge, depending on the resources that the person has to deal with the situation.

In addition to these more fundamental questions, measuring the physiological correlates of freezing might also be helpful in addressing more applied questions and finding ways to intervene in socially stressful situations. A relevant type of situation that comes to mind is unexpected social interactions (e.g., contact with counter-stereotypical individuals). Previous studies have shown that during such situations, people show physiological correlates of threat (Mendes et al., 2007). This may be partly explained by people initially freezing, which may subsequently lead to a relatively rigid and awkward start of the interaction. Via possible misunderstandings (e.g., rigid behavior is interpreted as prejudice), this could result in a vicious circle of a negative and threatening interaction. Another applied area where freezing can be relevant is decision-making where insights into the unfolding of freezing might be informative for the time people need to make thoughtful decisions in stressful situations (e.g., during conflict or after getting a threatening medical diagnosis, see Gladwin et al., 2016; Keinan & Friedland, 1987).

To conclude, the current research has largely replicated the freezing-to-social-threat effect initially demonstrated by Roelofs et al. (2010). We hope our work strengthens the confidence in the effect and paves the way for further research and applications in the context of (social) threat and physiology.
